# Near-infrared-responsive, superparamagnetic Au@Co nanochains

**DOI:** 10.3762/bjnano.8.168

**Published:** 2017-08-14

**Authors:** Varadee Vittur, Arati G Kolhatkar, Shreya Shah, Irene Rusakova, Dmitri Litvinov, T Randall Lee

**Affiliations:** 1 Department of Chemistry and the Texas Center for Superconductivity, University of Houston, 4800 Calhoun Road, Houston, TX 77204, USA; 2 Department of Physics and the Texas Center for Superconductivity, University of Houston, 4800 Calhoun Road, Houston, TX 77204, USA; 3 Department of Electrical and Computer Engineering, University of Houston, 4800 Calhoun Road, Houston, TX 77204, USA

**Keywords:** Au@Co, magneto-optical, nanochains, near-IR-active, superparamagnetic

## Abstract

This manuscript describes a new type of nanomaterial, namely superparamagnetic Au@Co nanochains with optical extinctions in the near infrared (NIR). The Au@Co nanochains were synthesized via a one-pot galvanic replacement route involving a redox-transmetalation process in aqueous medium, where Au salt was reduced to form Au shells on Co seed templates, affording hollow Au@Co nanochains. The Au shells serve not only as a protective coating for the Co nanochain cores, but also to give rise to the optical properties of these unique nanostructures. Importantly, these bifunctional, magneto-optical Au@Co nanochains combine the advantages of nanophotonics (extinction at ca. 900 nm) and nanomagnetism (superparamagnetism) and provide a potentially useful new nanoarchitecture for biomedical or catalytic applications that can benefit from both activation by light and manipulation using an external magnetic field.

## Introduction

The unique properties of magnetic nanoparticles have led to diverse applications in the fields of magnetic data storage, catalysis, magnetic fluids, biosensors, drug delivery, and magnetic imaging [1–6]. Considerable efforts have been taken to tailor the magnetic properties to suit specific applications [7–8]. In parallel, there have been significant advances in the development of plasmonic nanoparticles (e.g., gold and silver), and efforts to tune the extinction wavelength (e.g., into the near infrared (NIR)) are ongoing [9]. Plasmonic nanoparticles exhibit intense colors and have been used in various applications, such as energy conversion in solar cells [10–11], biosensing [12], photothermal therapy [13], and biomedical imaging [14]. Surface modification with an inorganic coating, such as silica, can lend biocompatibility to the nanoparticles [15–18]. A gold shell on magnetic nanoparticles not only renders the nanoparticles biocompatible but also gives rise to distinct optical properties [18–19]. Noble metal nanoparticles, such as gold and silver, possess the unique property of surface plasmon resonance (SPR); the latter exhibit a strong extinction band in the visible region [19–20]. Importantly, studies have focused on the synthesis of magnetic core–plasmonic shell structures to obtain versatile, hybrid nanoparticles with dual functionality [21–23].

The NIR region of light from approximately 800 to 1200 nm can penetrate human tissue (i.e., the ”water window”) and is minimally absorbed by tissue chromophores and water [24]. Therefore, tunable plasmonic nanoparticles that can respond to NIR light and can be manipulated with a magnetic field hold great promise. Among various magnetic nanoparticles, cobalt nanoparticles have attracted much interest due to their strong magnetic properties and their greater stability toward oxidation compared to Ni- and Fe-based magnetic nanoparticles [23].

Notably, there have been a handful of studies on magneto-optical nanostructures consisting of cobalt coated with gold. Bao and co-workers synthesized magnetic Co–Au core–shell nanoparticles [25] by reducing an organo-gold compound onto a cobalt seed with a weak reducing agent in toluene. These particles showed superparamagnetic behavior and a strong optical extinction at ca. 680 nm. Similarly, Wetz and co-workers prepared hybrid Co–Au nanorods via decomposition of an organometallic compound under hydrogen [26]. The particles showed extinction maxima at ca. 550 nm and ca. 720 nm. Liang et al. used Co nanoparticles as sacrificial templates to prepare hollow gold nanospheres [27]. The formation of these particles involved a redox-transmetalation process between the core and shell arising from the difference between their reduction potentials. Cobalt cores were oxidized by H^+^ from aqueous HAuCl_4_ solution until Co nanoparticles were completely consumed, which led to the formation of the hollow gold nanostructures. The surface plasmon resonance of these particles appeared at 628 nm.

In addition to these studies on gold-coated cobalt nanoparticles, there are, to our knowledge, only three reports of gold-coated cobalt (Au@Co) nanochains. Huang and co-workers synthesized bimetallic PtCo and AuCo magnetic nanochains, and showed how the magnetic and electronic properties could be modulated depending on the noble metal selected [28]. Duan et al. synthesized Pt-coated Co nanochains in an aqueous medium by magnetic-field induced assembly and galvanic replacement reaction, and Lu et al. synthesized Au-coated Co nanochains in a similar fashion [29–30]. However, none of these studies explored the optical properties of the Au-coated magnetic Co nanochains. Consequently, there have been no reports characterizing both the optical and the magnetic properties of this unique class of nanomaterial.

**Scheme 1:**
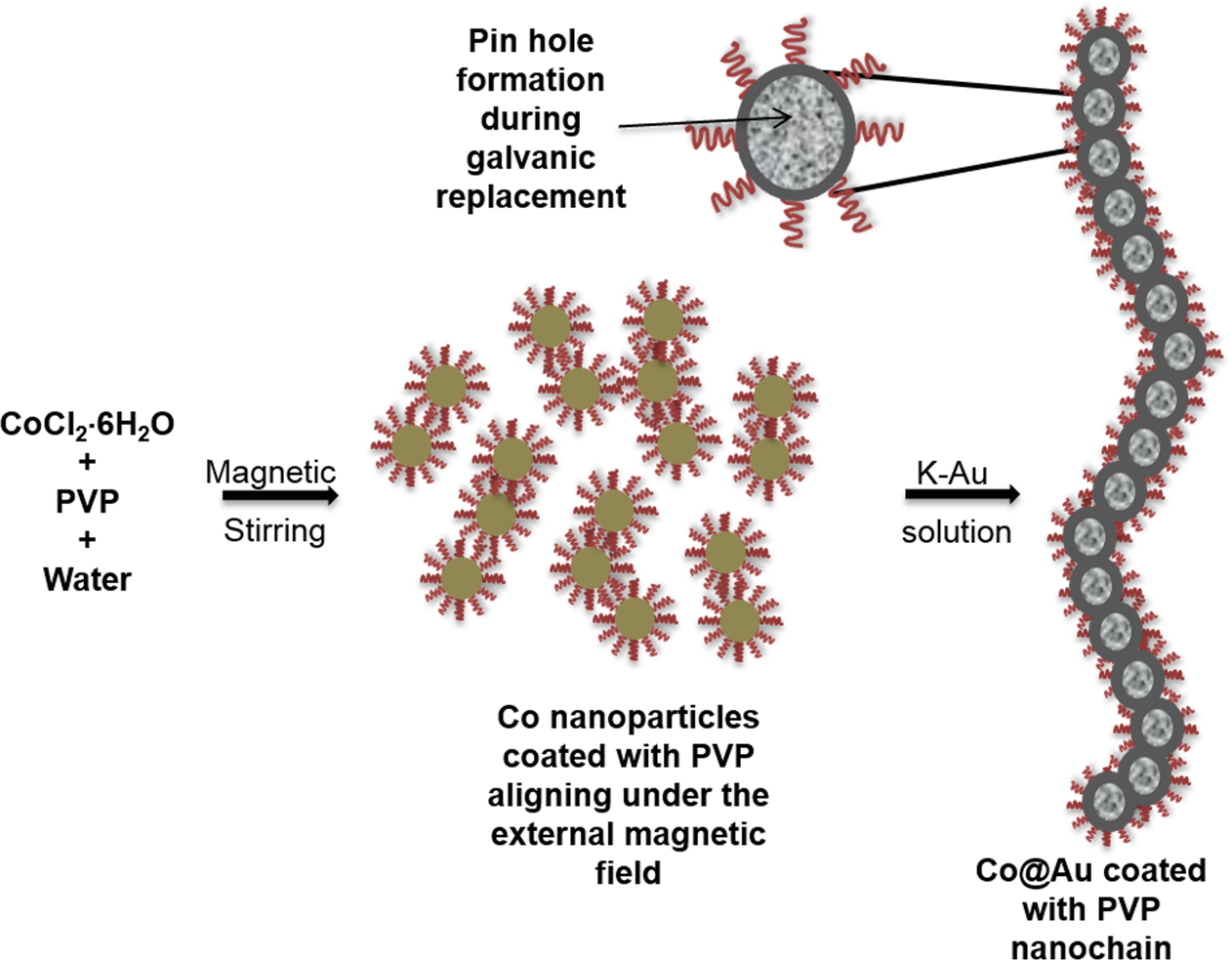
Synthesis of Au@Co nanochains.

In the present investigation, we describe a one-pot synthesis of magnetic Au@Co nanochains using a wet-chemical method based on a galvanic replacement reaction. Scheme 1 shows that cobalt nanoparticles serve as seed templates over which Au salt is chemically reduced into a shell. By adding Au stock solution to suspensions of the cobalt core solution, and with the assistance of polyvinylpyrrolidone (PVP), Au@Co nanochains with a hollow interior are formed. Using this new methodology, we were able to shift the optical extinction of the generated nanoparticles to the near-infrared (NIR) region (about 900 nm), which is significantly higher than the maximum of 700 nm observed previously for related nanostructures [28–30]. This considerable increase can be attributed to the formation of both the hollow interior and the chain structure in the present materials.

Previous research on Au and Ag nanochains demonstrated that the plasmon resonance of two interacting particles undergoes a red shift from that of a single particle because of near-field coupling. This shift in wavelength is proportional to the number of nanoparticles in the chain [31–32]. These new types of magnetic nanostructures find use in several applications, such as combined MRI imaging and photothermal treatment and bio-separation, that require magnetic properties and strong optical extinctions in the visible and/or near-infrared [33–35]. Our Au@Co nanochains are superparamagnetic at room temperature (blocking temperature of 150 K) and thus offer the capacity for control and delivery under the directed influence of an external magnetic field. Furthermore, the NIR-responsive capability (optical extinction maximum at ca. 900 nm) combined with their magnetic properties can plausibly be harnessed in nanotechnology-based biomedical applications, such as photo-thermally modulated drug delivery, photonic devices, and photo-thermal cancer therapy [10–13].

## Results and Discussion

### Morphology and composition of the Au@Co nanochains

**Figure 1:**
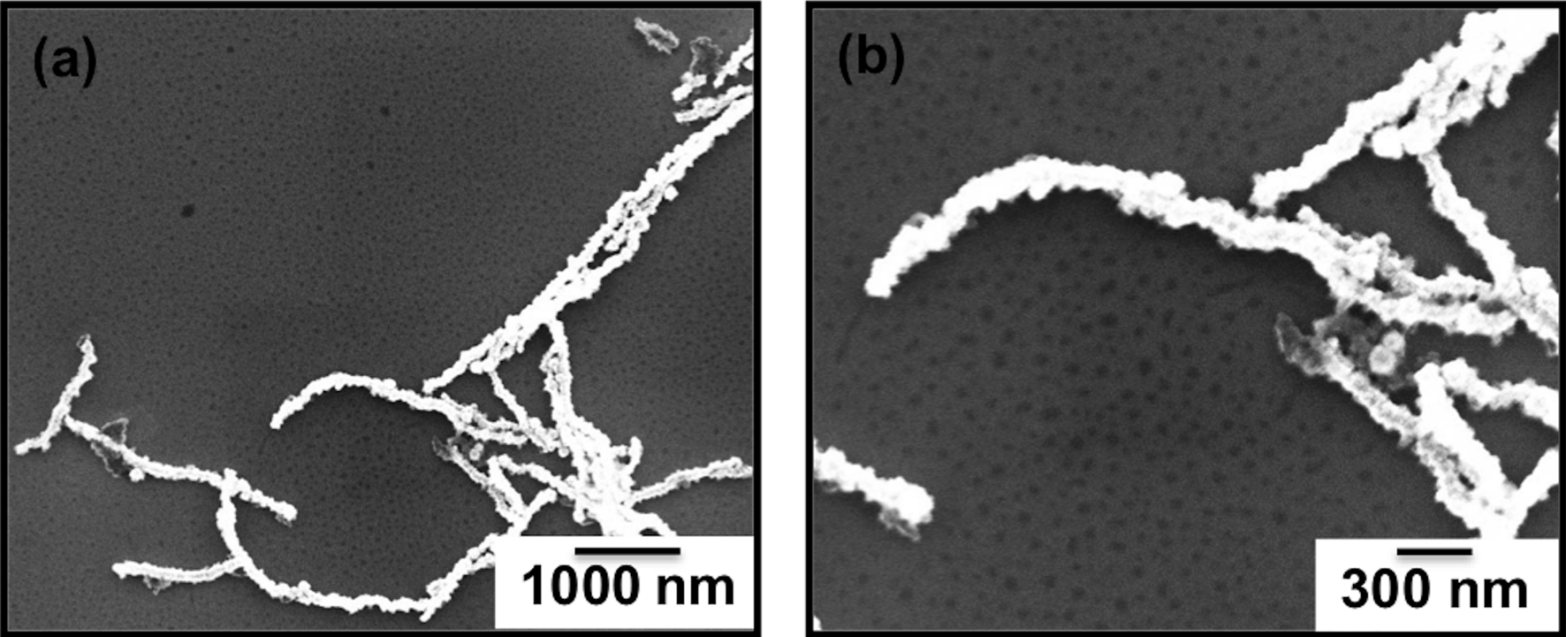
SEM images of the Au@Co nanochains at (a) low magnification and (b) high magnification.

**Figure 2:**
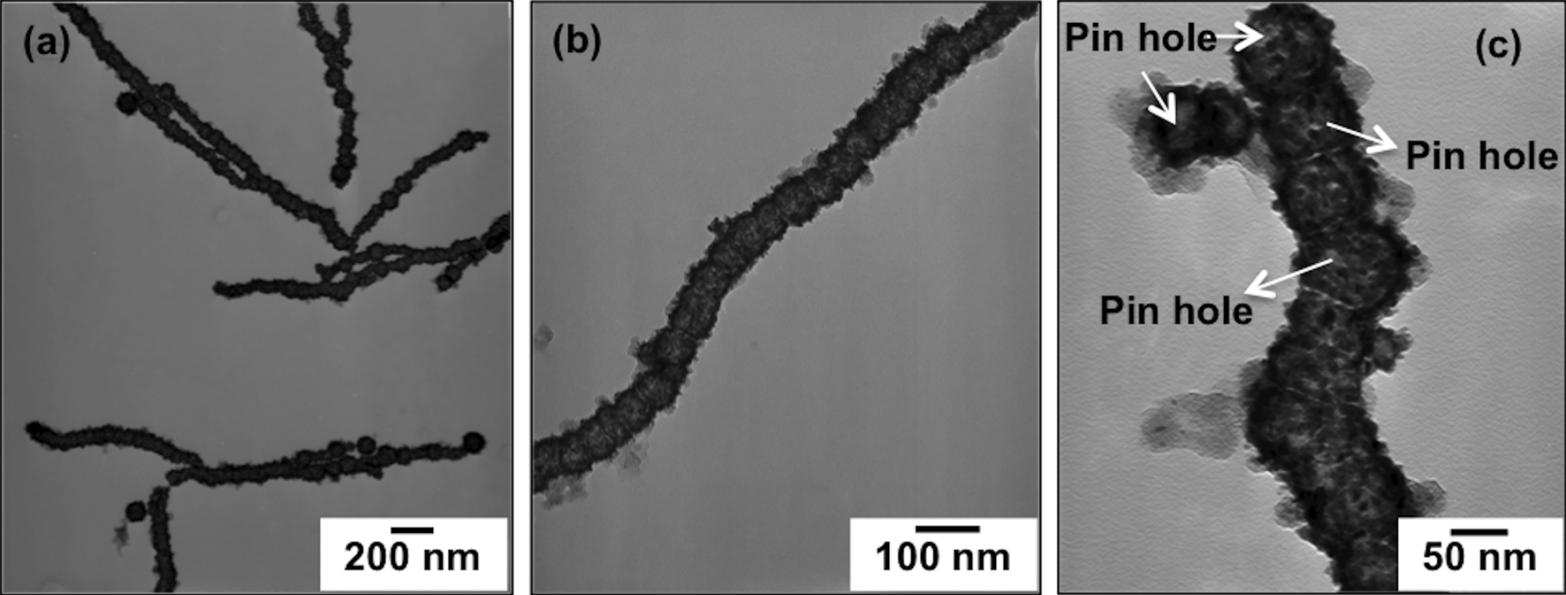
TEM images of the Au@Co nanochains: (a,b) low-magnification and (c) high magnification.

The synthesis of the Au@Co nanochains proceeded using wet-chemical methods via a galvanic replacement reaction [27]. Cobalt nanoparticles were used as templates for the nanochain synthesis. Because the standard reduction potentials of the AuCl_4_^−^/Au pair (*E* = 0.990 V vs the standard hydrogen electrode, SHE) is higher than that of the Co^2+^/Co pair (*E* = −0.277 V vs SHE), AuCl_4_^−^ will be reduced to Au(0) upon addition of the basic solution of potassium and gold (K–gold solution) to the Co nanoparticle stock solution, leading to the formation of Au@Co. The structure and morphology of the Au@Co nanochains were characterized by using transmission electron microscopy (TEM) and scanning electron microscopy (SEM) as shown in Figure 1 and Figure 2, where sub-micrometer-sized chains are readily apparent.

Figure 2 illustrates the difference in contrast between the inner and outer regions of the chains, consistent with the growth of a thin gold shell around the Co nanoparticles. The high-magnification TEM images also show that the chains are composed of distinct spherical nanoparticles, with an average outer diameter of 75 ± 3 nm, in close contact with each other; the shell is rough, with a typical thickness of about 14 ± 6 nm. Moreover, some pinholes were observed in the wall of the bimetallic nanochains, indicating the formation of a hollow nanostructure as shown in Figure 2c.

**Figure 3:**
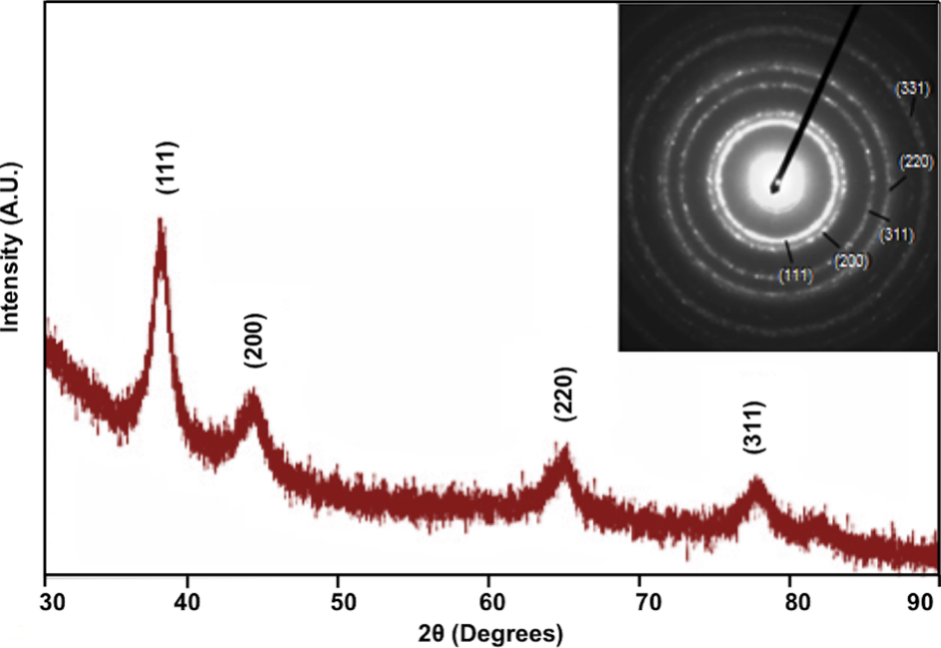
X-ray diffraction pattern and SAED pattern (inset) for the Au@Co nanochains.

Figure 3 provides the X-ray diffraction (XRD) data of the synthesized nanochains. The XRD pattern shows peaks at 2θ = 38.34°, 46.36°, 65.24° and 77.11°, which correspond, respectively, to the (111), (200), (220), and (311) crystallographic planes of the face-centered cubic (fcc) structure of gold. The selected area electron diffraction (SAED) shown in the inset is also consistent with a single-phase fcc structure [36].

**Figure 4:**
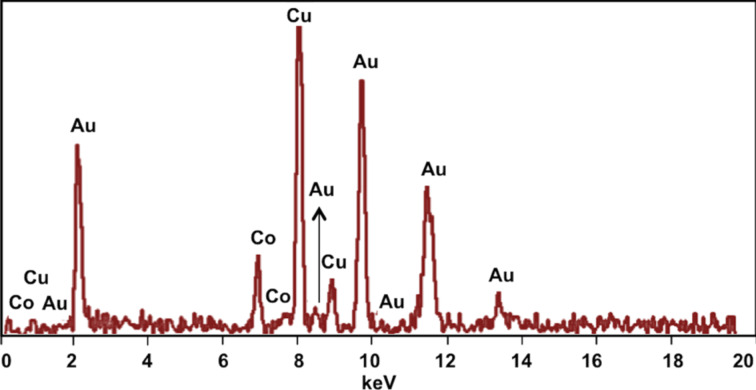
EDX spectrum of the Au@Co nanochains.

The chemical composition of the bimetallic nanochains was evaluated by energy dispersive X-ray spectroscopy (EDX) and X-ray photoelectron spectroscopy (XPS). Figure 4 shows the EDX spectrum with peaks characteristic of gold (Mα, Lα, Lβ, and Lγ) at 2.12, 9.71, 11.60, and 13.45 keV, respectively, and peaks characteristic of Co (Lα and Kα) at 0.78 and 6.93 keV, respectively. Peaks from the supporting grid of copper are observed at 8.04 and 8.90 keV (Kα and Kβ), respectively. The peak intensity is related to the abundance of each element, and the EDX data averaged over multiple runs confirm that the approximate stoichiometry of the nanochains is Co_27_Au_72_.

**Figure 5:**
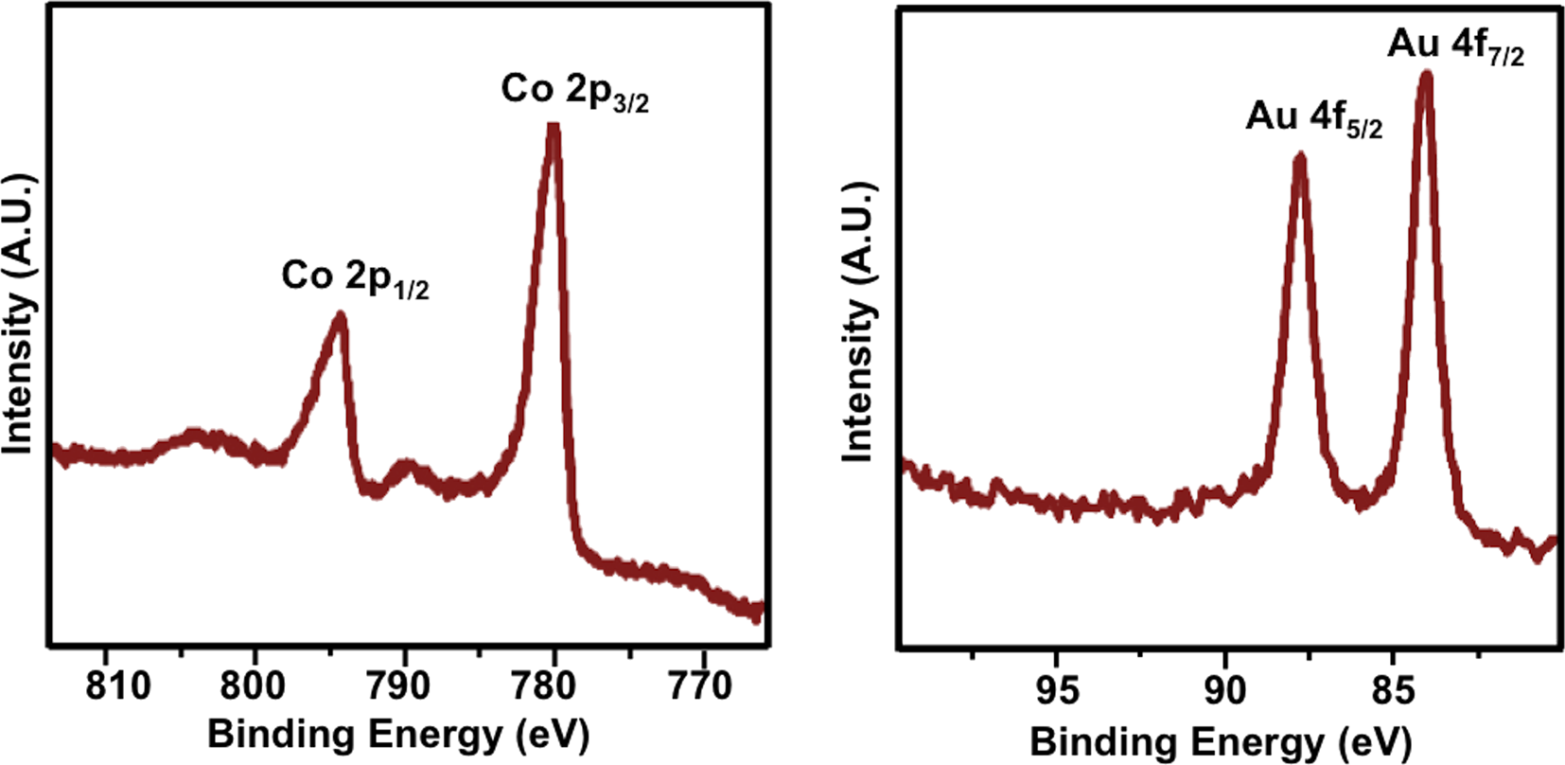
XPS spectra of the Au@Co nanochains.

We also used XPS to analyze the chemical composition of the nanochains (Figure 5). The XPS spectrum shows two peaks at 84.0 and 87.7 eV, which can be attributed to Au 4f electrons, as well as peaks at 780.2 and 795.3 eV, which can be attributed to Co 2p electrons. The minor broadened peaks that were observed at binding energies of ca. 790 eV and just above 800 eV can be attributed to cobalt oxide [37]. Since XPS is a surface technique, the penetration depth is less than 10 nm [38]; therefore, we would not expect to see Co in these analyses. However, XPS studies of our gold-coated cobalt nanochains showed peaks for Co 2p_1/2_ electrons (793 eV) and Co 2p_3/2_ electrons (778 eV). We believe that the observation of Co by XPS was due to the existence of pinholes and/or the hollow structure of the particles, which is consistent with the TEM images.

Reports of the formation of hollow nanostructures via galvanic replacement are known [39–46]. There are also some examples where Co nanoparticles are used as templates [24,27,47–48]. By varying the surfactant added, we were able to obtain Au-coated Co nanochains. As the standard reduction potentials for the AuCl_4_^−^/Au pair is much higher than that of the Co^2+^/Co pair, Co nanoparticles were immediately oxidized to Co^2+^ when the K–gold solution was added. Given the rapid nature of this replacement reaction, it is likely that nucleation of the reduced Au atoms led to small Au particles that eventually grew into a thin Au shell covering the cobalt nanoparticles. At the same time, because of its extended polymeric chain structure, PVP, which was present as a colloidal stabilizer, behaved as a soft template for chain growth [47], as described below. Notably, the shells appear to possess varying porosity. Consequently, it is plausible that Co^2+^ and AuCl_4_^−^ ions continuously diffused through the shell during the galvanic replacement reaction and thereby gave rise to the formation of the hollow nanostructure.

**Figure 6:**
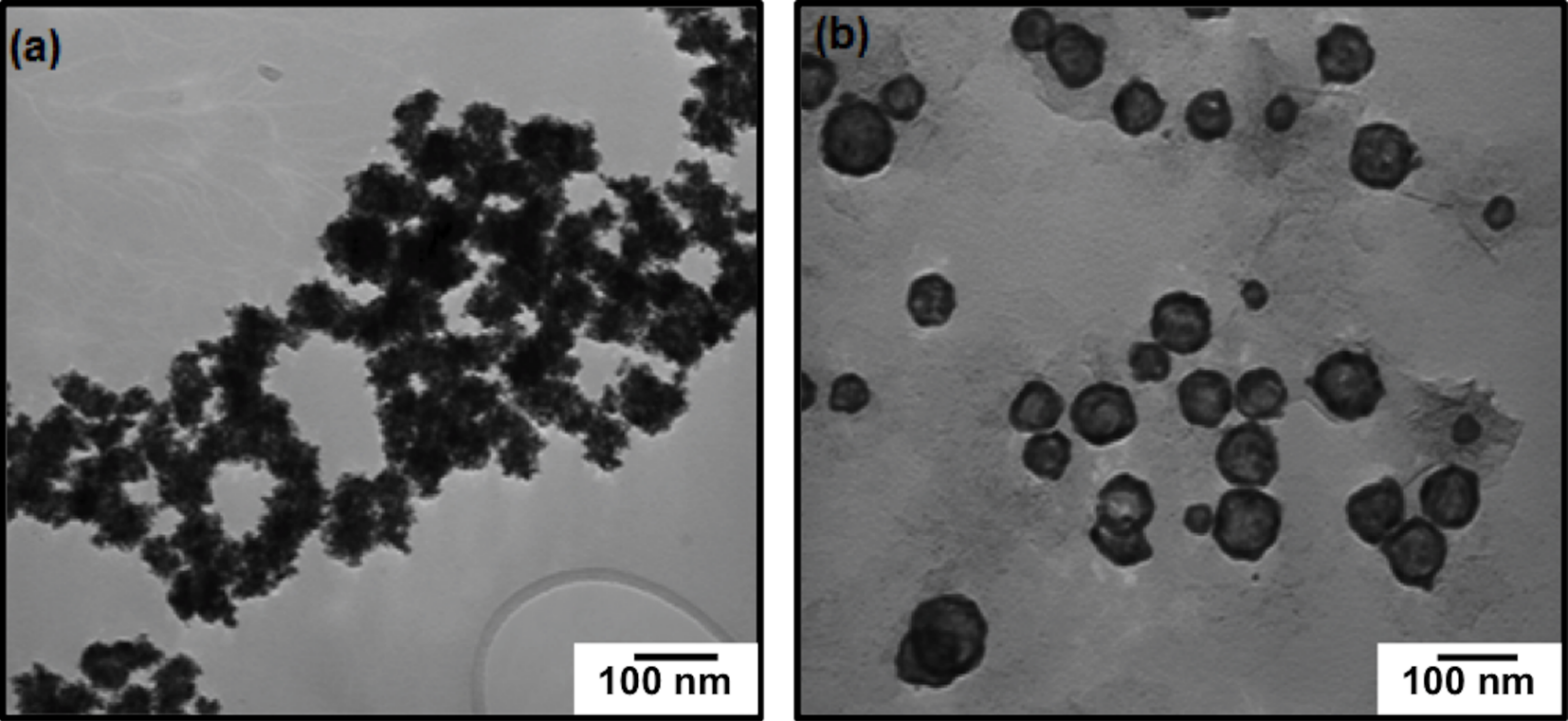
TEM images of Au@Co magnetic nanoparticles prepared (a) in the absence of PVP and (b) in the absence of a magnetic stirrer.

As noted above, PVP is widely used as a colloidal stabilizer to inhibit the aggregation of metal nanoparticles such as gold [49–52], silver [53–54], platinum [55–57], palladium [46,57–59], nickel [60–61], and cobalt [62–63]. To verify this hypothesis in our system, we conducted the synthetic steps under similar conditions without adding any PVP. In the absence of PVP, the Co nanoparticles aggregated, leading to the formation of irregularly shaped particles as shown in Figure 6a. Additionally, we believe that PVP plays an important role in the formation of the chain structure; indeed, there have been some reports describing the formation of gold and nickel nanochains using PVP as a colloidal stabilizer [60,64–65]. Furthermore, in our experiments, we noted that the presence of the magnetic stirrer was also critical for the formation of the nanochains. Additional experiments to confirm the contribution of the magnetic stirrer were performed by swirling the solution by hand rather than using a magnetic stirrer. Figure 6b shows the TEM image of Au@Co nanoparticles prepared without using a magnetic stirrer. The TEM image shows discrete Au@Co nanospheres in contrast to the chain-linked structures obtained using a magnetic stirrer. This control experiment indicates a likely interaction between the magnetic nanoparticles and the external magnetic field produced by the magnetic stirrer during the galvanic replacement reaction. We hypothesize that both PVP and the use of a magnetic stirrer contribute to the assembly of these magnetic nanoparticles into nanochains.

### Optical properties of the Au@Co nanochains

**Figure 7:**
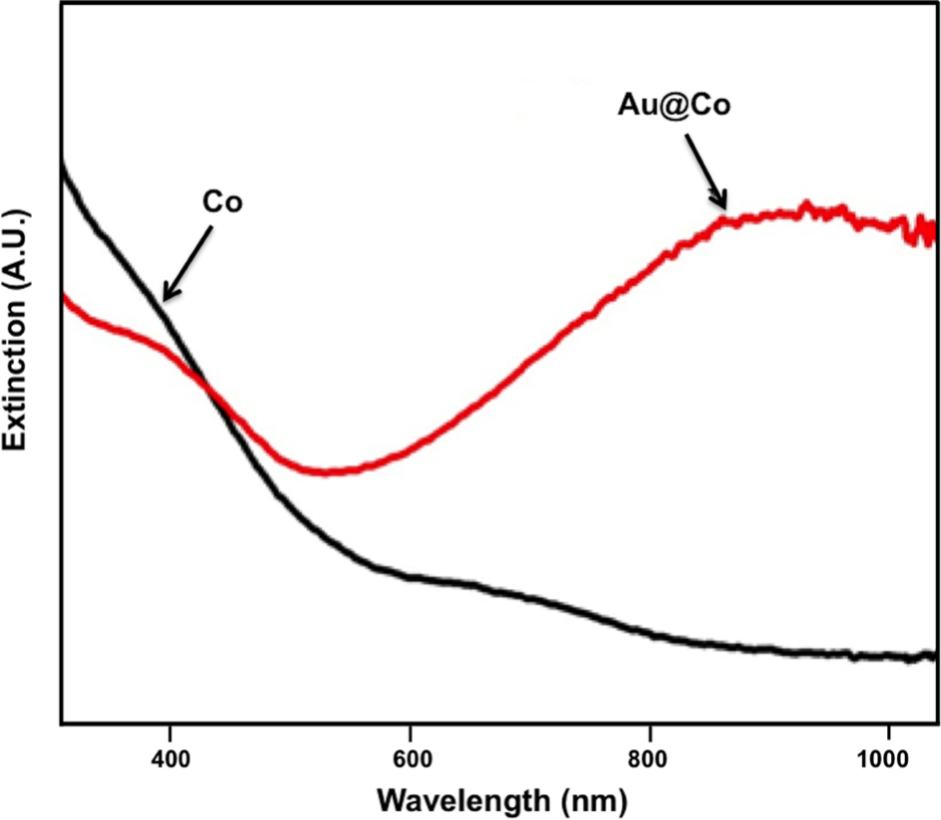
Extinction spectra of (a) Co nanoparticles and (b) Au@Co nanochains.

The optical properties of the Au@Co nanochains were characterized by UV–vis spectroscopy. Figure 7 shows the characteristic extinction spectrum of the Co nanoparticle solution before and after adding the K–gold solution. No plasmon resonance peak was observed for the bare cobalt nanoparticles, and the extinction decreased with an increase in the wavelength as expected based on reports in the literature [66–67]. However, upon the addition of K–gold solution, a plasmon resonance appeared as a strong peak at ca. 900 nm; importantly, the nanochains possess no extinction bands characteristic of either bare gold nanoparticles or pure gold nanochains [68–70]. As mentioned above, Au-coated hollow nanospheres using Co nanoparticles as a template exhibited a plasmonic resonance at 628 nm [27]. Consequently, our results represent a significant and potentially useful outcome, as there have been, to our knowledge, no prior reports of NIR-responsive nanochain systems. There is a strong need for nanomaterials that are NIR-active [10–12,14], and our Au@Co nanochains help to fill in this need.

### Magnetic properties of the Au@Co nanochains

**Figure 8:**
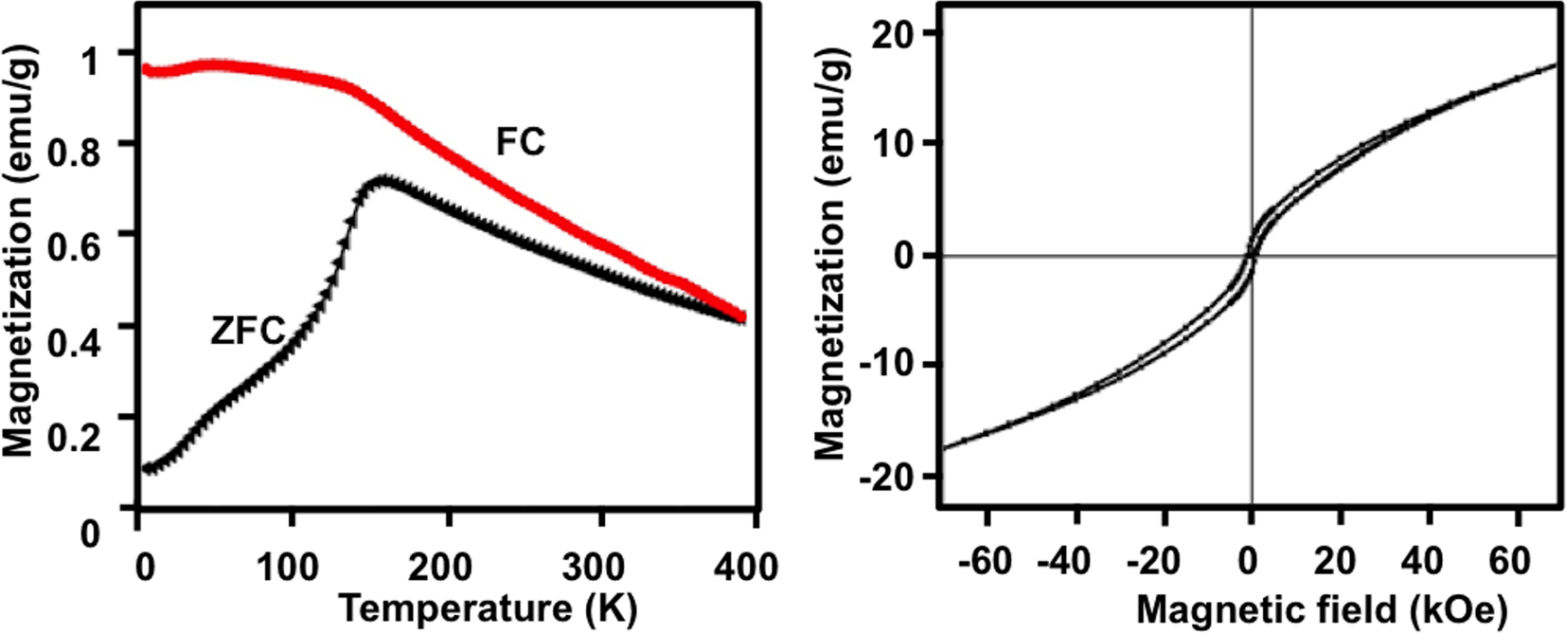
Magnetic properties of the Au@Co nanochains: (a) zero-field-cooling (ZFC), field-cooled (FC) at 100 Oe applied field, and (b) field-dependent magnetization (*M* vs *H*) hysteresis loop.

In addition to their NIR extinction, our dual-functionality Au@Co nanochains possess magnetic properties, which were evaluated using a superconducting quantum interference device (SQUID) magnetometer. The temperature dependence of the magnetization for nanochains was measured in an applied field of 100 Oe from 0 to 400 K. Figure 8a shows the zero-field-cooling (ZFC) and field-cooled (FC) measurements of the Au@Co nanochains. The sharp maximum in the ZFC curve demonstrated the blocking behavior of the nanochains, with a blocking temperature of 150 K [71]. The field-dependent magnetization was measured at 5 K and is shown in Figure 8b; the magnetization is above 15 emu/g at a field of 60 kOe. No saturation was observed in the magnetization at 5 K. The absence of a clear saturation of the Au@Co nanochains at low temperatures and high external fields, as seen in Figure 8b, suggests the presence of paramagnetism or a paramagnetic-like phase in the material. On the whole, the magnetic behavior observed in Figure 8a,b is typical for superparamagnetic materials and suggests that the Au@Co nanochains are superparamagnetic.

## Conclusion

This study reports the synthesis and characterization of gold-coated superparamagnetic Co nanochains with NIR extinction (ca. 900 nm). We demonstrated the synthesis of magneto-optical Au@Co nanochains using a modified wet-chemical method based on a galvanic replacement reaction and highlighted the significant roles of PVP and magnetic stirring in the formation of the chain structure. The Au@Co nanochains possess a strong optical extinction in the NIR spectral region, making them useful for several applications such as photo-thermally modulated drug delivery, photonic devices, and photo-thermal cancer therapy. In addition, these nanochains are superparamagnetic at room temperature, which offers the capacity for manipulation under an external magnetic field. Because of their dual magneto-optical properties, our Au@Co nanochains can be used for both delivery and treatment, making them a unique new choice for biomedical applications in which such dual functional properties can be utilized.

## Experimental

### Materials

Cobalt chloride (Baker), polyvinylpyrrolidone (*M*_W_ ≈ 50000, Aldrich), sodium borohydride (Aldrich), potassium carbonate (Aldrich), hydrogen tetrachloroaurate(III) hydrate (Strem), nitric acid (EM Science) and hydrochloric acid (EM Science) were purchased from the indicated suppliers and used without purification. Water was purified to a resistivity of 18 MΩ·cm (Academic Milli-Q Water System; Millipore Corporation). All glassware and equipment was cleaned using an aqua regia solution (HCl/HNO_3_ 3:1), thoroughly rinsed with Milli-Q water, and then dried prior to use. A basic solution of potassium and gold (K–gold solution) was prepared under vigorous stirring by adding 2 mL of 1% HAuCl_4_·H_2_O solution to 100 mL of Milli-Q water containing 0.025 g of potassium carbonate (K_2_CO_3_). The mixture changed from yellow to colorless after the reaction had proceeded for an additional 30 min. The K–gold solution was stored under refrigeration in the dark for two days (with help of aluminum foil) until use.

### Synthesis of the Au@Co nanochains

The synthesis of the nanochains proceeded using a wet-chemical method in which a reactor was charged with 8.5 mg of CoCl_2_·6H_2_O, 100 mg PVP, and 50 mL of water under vigorous magnetic stirring (IKAMAG^®^ C-Mag HS 7). The reaction was purged with Ar for 15 min and then a freshly prepared 5 mL aqueous solution of 0.013 M NaBH_4_ was added dropwise. A light brown color was observed in the solution, indicating the formation of small cobalt nanoparticles [27]. An aliquot (5 mL) of K–gold solution was immediately added into the cobalt nanoparticle solution; subsequently, the color of the mixture changed from light brown to dark blue. The mixture was collected by centrifugation at 3000 rpm for 30 min using an RC-3B refrigerated centrifuge (Sorvall Instruments) and redispersed in Milli-Q water prior to analysis.

### Characterization methods

The size and morphology of the composite nanochains were characterized by field-emission scanning electron microscopy (FE-SEM) and TEM. FE-SEM measurements were carried out using a JEOL JSM 6330F instrument operating at an accelerating voltage of 15 kV. TEM measurements were carried out using a JEOL JEM-2000 FX electron microscope operating at an accelerating voltage of 200 kV. The samples were prepared by placing small drops of solution containing the dispersed nanochains on a silicon wafer (for FE-SEM) or on 300-mesh holey carbon-coated copper grids (for TEM) and allowing the solvent to evaporate.

Analysis by XRD was performed using a Siemens D-5000 with monochromatic Cu Kα radiation (λ = 1.540562 Å), and EDX measurements were collected using an Oxford EDX attached to the TEM microscope to confirm the structure. All extinction spectra were recorded at room temperature on a Cary 50 Scan UV–visible spectrometer over the wavelength range of 250–1100 nm. The magnetic properties of the nanochains were obtained using a SQUID magnetometer with fields up to 5 T. The temperature-dependent magnetization curves varying between 5 and 400 K were measured in an applied magnetic field of 100 Oe.

## References

[1] Berry C C, Curtis A S G (2003). J Phys D: Appl Phys.

[2] Grancharov S G, Zeng H, Sun S, Wang S X, O'Brien S, Murray C B, Kirtley J R, Held G A (2005). J Phys Chem B.

[3] Tartaj P, del Puerto Morales M, Veintemillas-Verdaguer S, González-Carreño T, Serna C J (2003). J Phys D: Appl Phys.

[4] Ji X, Shao R, Elliott A M, Stafford R J, Esparza-Coss E, Bankson J A, Liang G, Luo Z-P, Park K, Markert J T (2007). J Phys Chem C.

[5] Jeong U, Teng X, Wang Y, Yang H, Xia Y (2007). Adv Mater.

[6] Alivisatos A P (1996). Science.

[7] Schmid G (1994). Gluster and Colloids: From Theory to Applications.

[8] Kolhatkar A G, Jamison A C, Litvinov D, Willson R C, Lee T R (2013). Int J Mol Sci.

[9] Jain P K, Xiao Y, Walsworth R, Cohen A E (2009). Nano Lett.

[10] Linic S, Christopher P, Ingram D B (2011). Nat Mater.

[11] Clavero C (2014). Nat Photonics.

[12] Howes P D, Chandrawati R, Stevens M M (2014). Science.

[13] Shanmugam V, Selvakumar S, Yeh C-S (2014). Chem Soc Rev.

[14] Dreaden E C, Alkilany A M, Huang X, Murphy C J, El-Sayed M A (2012). Chem Soc Rev.

[15] Yi D K, Selvan S T, Lee S S, Papaefthymiou G C, Kundaliya D, Ying J Y (2005). J Am Chem Soc.

[16] Santra S, Tapec R, Theodoropoulou N, Dobson J, Hebard A, Tan W (2001). Langmuir.

[17] Singh R K, Kim T-H, Patel K D, Knowles J C, Kim H-W (2012). J Biomed Mater Res, Part A.

[18] Malvindi M A, De Matteis V, Galeone A, Brunetti V, Anyfantis G C, Athanassiou A, Cingolani R, Pompa P P (2014). PLoS One.

[19] Mayer K M, Hafner J H (2011). Chem Rev.

[20] Haes A J, Van Duyne R P (2004). Expert Rev Mol Diagn.

[21] Levin C S, Hofmann C, Ali T A, Kelly A T, Morosan E, Nordlander P, Whitmire K H, Halas N J (2009). ACS Nano.

[22] Xu Z, Hou Y, Sun S (2007). J Am Chem Soc.

[23] Alù A, Engheta N (2009). Opt Express.

[24] Weissleder R (2001). Nat Biotechnol.

[25] Bao Y, Calderon H, Krishnan K M (2007). J Phys Chem C.

[26] Wetz F, Soulantica K, Falqui A, Respaud M, Snoeck E, Chaudret B (2007). Angew Chem, Int Ed.

[27] Liang H-P, Wan L-J, Bai C-L, Jiang L (2005). J Phys Chem B.

[28] Huang W-F, Zhang Q, Zhang D-F, Zhou J, Si C, Guo L, Chu W-S, Wu Z-Y (2013). J Phys Chem C.

[29] Duan S, Wang R (2013). Prog Nat Sci: Mater Int.

[30] Lu Y, Zhao Y, Yu L, Dong L, Shi C, Hu M-J, Xu Y-J, Wen L-P, Yu S-H (2010). Adv Mater.

[31] Su K-H, Wei Q-H, Zhang X, Mock J J, Smith D R, Schultz S (2003). Nano Lett.

[32] Zhu S-Q, Zhang T, Guo X-L, Shan F, Zhang X-Y (2014). J Nanomater.

[33] Larson T A, Bankson J, Aaron J, Sokolov K (2007). Nanotechnology.

[34] Lim J, Tilton R D, Eggeman A, Majetich S A (2007). J Magn Magn Mater.

[35] Peng S, Lei C, Ren Y, Cook R E, Sun Y (2011). Angew Chem, Int Ed.

[36] Song Y, Modrow H, Henry L L, Saw C K, Doomes E E, Palshin V, Hormes J, Kumar C S S R (2006). Chem Mater.

[37] Barreca D, Massignan C, Daolio S, Fabrizio M, Piccirillo C, Armelao L, Tondello E (2001). Chem Mater.

[38] Vickerman J C (1997). Surface Analysis - The Principle Techniques.

[39] Sun Y, Xia Y (2004). J Am Chem Soc.

[40] Sun Y, Xia Y (2003). Nano Lett.

[41] Jin Y, Dong S (2003). J Phys Chem B.

[42] Srnová-Šloufová I, Lednický F, Gemperle A, Gemperlová J (2000). Langmuir.

[43] Sun Y, Mayers B T, Xia Y (2002). Nano Lett.

[44] Gao J, Ren X, Chen D, Tang F, Ren J (2007). Scr Mater.

[45] Chen J, Wiley B, McLellan J, Xiong Y, Li Z-Y, Xia Y (2005). Nano Lett.

[46] Xiong Y, Wiley B, Chen J, Li Z-Y, Yin Y, Xia Y (2005). Angew Chem, Int Ed.

[47] Guo L, Liang F, Wen X, Yang S, He L, Zheng W, Chen C, Zhong Q (2007). Adv Funct Mater.

[48] Vasquez Y, Sra A K, Schaak R E (2005). J Am Chem Soc.

[49] Cheng H-W, Huan S-Y, Wu H-L, Shen G-L, Yu R-Q (2009). Anal Chem.

[50] Nakazato Y, Taniguchi K, Ono S, Eitoku T, Katayama K (2009). Phys Chem Chem Phys.

[51] Mishra A, Tripathy P, Ram S, Fecht H-J (2009). J Nanosci Nanotechnol.

[52] Tan Y, Dai X, Li Y, Zhu D (2003). J Mater Chem.

[53] Pietrobon B, Kitaev V (2008). Chem Mater.

[54] Tsuji T, Thang D-H, Okazaki Y, Nakanishi M, Tsuboi Y, Tsuji M (2008). Appl Surf Sci.

[55] Bonet F, Delmas V, Grugeon S, Herrera Urbina R, Silvert P-Y, Tekaia-Elhsissen K (1999). Nanostruct Mater.

[56] Park J Y, Aliaga C, Renzas J R, Lee H, Somorjai G A (2009). Catal Lett.

[57] Teranishi T, Hori H, Miyake M (1997). J Phys Chem B.

[58] Ayyappan S, Srinivasa Gopalan R, Subbanna G N, Rao C N R (1997). J Mater Res.

[59] Teranishi T, Miyake M (1998). Chem Mater.

[60] Liu X, Guo M, Zhang M, Wang X, Guo X, Chou K (2008). Rare Met.

[61] Couto G G, Klein J J, Schreiner W H, Mosca D H, de Oliveira A J A, Zarbin A J G (2007). J Colloid Interface Sci.

[62] Kato Y, Sugimoto S, Shinohara K-i, Tezuka N, Kagotani T, Inomata K (2002). Mater Trans.

[63] Platonova O A, Bronstein L M, Solodovnikov S P, Yanovskaya I M, Obolonkova E S, Valetsky P M, Wenz E, Antonietti M (1997). Colloid Polym Sci.

[64] Zhou W, He L, Cheng R, Guo L, Chen C, Wang J (2009). J Phys Chem C.

[65] Zhang D-F, Niu L-Y, Jiang L, Yin P-G, Sun L-D, Zhang H, Zhang R, Guo L, Yan C-H (2008). J Phys Chem C.

[66] Petit C, Pileni M P (1997). J Magn Magn Mater.

[67] Podlaha E J, Henry L, Guo Z (2006). ECS Trans.

[68] El-Sayed I H, Huang X, El-Sayed M A (2005). Nano Lett.

[69] Hussain I, Brust M, Barauskas J, Cooper A I (2009). Langmuir.

[70] Polavarapu L, Xu Q-H (2008). Langmuir.

[71] Chen D, Liu S, Li J, Zhao N, Shi C, Du X, Sheng J (2009). J Alloys Compd.

